# Bacterial effectors targeting the host ubiquitin system

**DOI:** 10.1042/BST20260089

**Published:** 2026-10-29

**Authors:** Paula Eschger, Thomas Hermanns, Kay Hofmann

**Affiliations:** Institute for Genetics, University of Cologne, Zülpicher Straße 47a, D-50674 Cologne, Germany

**Keywords:** autophagy, host-pathogen interactions, ubiquitin ligases, ubiquitins

## Abstract

Intracellular bacteria are largely shielded from humoral immunity but remain vulnerable to cell-autonomous defense mechanisms, many of which depend on the ubiquitin system. To evade autophagy and lysosomal targeting, numerous bacteria have evolved secreted effectors that disrupt or manipulate the host ubiquitin machinery to their advantage. Bacterial deubiquitinases can protect bacterial surfaces or their enclosing vacuoles from ubiquitin coating and interfere with host inflammatory and cell death pathways. Conversely, bacterial ubiquitin ligases may induce the degradation of host proteins, engaging in an evolutionary arms race with host ligases. In addition, ubiquitin-modifying and ubiquitin-crosslinking effectors can disable major host defense signaling pathways. In the present overview, we summarize currently known ubiquitin-directed effectors, their targets, and their roles in bacteria–host interaction.

## Introduction

Ubiquitination, the covalent attachment of the small protein ubiquitin to a substrate, is a versatile post-translational modification that regulates numerous cellular processes, including protein and organellar quality control, vesicular trafficking, inflammation, and the defense against pathogens. The transfer of ubiquitin onto a substrate involves a multi-step enzyme cascade, comprising ubiquitin activation (E1), conjugation (E2), and ligation (E3) steps [1]. The human genome encodes several hundred distinct ubiquitin ligases, which catalyze the final, substrate-specific E3 reaction. The complexity of the ubiquitin signaling system is further enhanced by the formation of diverse ubiquitin chain types [2]. These chains are generated through the conjugation of ubiquitin to one of the lysine residues or the N-terminus of another ubiquitin molecule, typically one that is already attached to a substrate. In addition to eight known homotypic chains, mixed and branched ubiquitin chains have also been identified [1]. In general, ubiquitin modification influences the fate of the targeted protein or structure, with the specific effect determined by the type of ubiquitination. Proteins linked with longer ubiquitin chains through lysines K48 or K11 are directed toward proteasomal degradation. Conversely, K63-linked chains facilitate the endocytosis and/or vesicular transport of membrane proteins to the lysosome. In non-membrane contexts, K63-linked chains and linear (M1-linked) chains play a role in regulating inflammation signaling and cell death. Additionally, multiple monoubiquitination events and various other chain types can modulate vesicular trafficking to the lysosome or target cellular cargoes for autophagic degradation.

Bacteria with an intracellular lifestyle can be targeted by ubiquitin-mediated defense pathways [3]. Once they enter a cell through phagocytosis or triggered endocytosis, some bacteria remain and multiply within membrane-bound organelles known as bacteria-containing vacuoles (BCVs) or inclusions, while others escape to live freely within the host cytoplasm. Among intracellular bacteria, *Legionella* and *Chlamydia*-like species replicate within membrane-bound vacuoles, whereas *Shigella, Listeria*, and pathogenic *Burkholderia* species typically escape into and replicate within the host cytosol; *Salmonella enterica* can adopt both vacuolar and cytosolic lifestyles [4]. Both cytoplasmic bacteria and BCV membranes are subject to ubiquitination, marking them for autophagic removal (xenophagy) or for the endolysosomal pathway [5]. Various host ligases and linkage types are involved in these processes; a well-characterized example is the initial ubiquitination of bacterial surface LPS by RNF213, followed by chain elongation through other ligases [6–8]. Beyond its role in directly targeting bacteria and BCVs, ubiquitination is also crucially involved in signaling pathways that orchestrate inflammation, cell death, and other defense responses [9,10].

Many intracellular bacteria secrete specialized effector proteins into the host cytoplasm, which can either incapacitate ubiquitin-based defense pathways or manipulate the host ubiquitin system to benefit the pathogen [3]. Bacteria residing within BCVs or those that have not (yet) entered the host cell can employ secretion systems such as T3SS, T4SS, and T6SS to translocate proteins across both bacterial and host membranes [11]. Bacteria directly exposed to the cytoplasm might also use simpler secretion mechanisms, as they do not need to cross a host-derived membrane. The bacterial effector repertoires exhibit a wide range of ubiquitin-directed activities. Traditionally, they are classified according to their molecular function as deubiquitinases (DUBs), ubiquitin ligases, ubiquitin modifiers, or ubiquitin-pathway modifiers. This classification is available for all effectors covered in the present review and is usually based on enzymatic assays performed with purified proteins, making it relatively unambiguous. Alternatively, effectors can be classified according to the biological pathways they target. For example, the prevention of ubiquitin marks on the bacterial or BCV surface can be achieved by their removal through the action of DUBs, by preventing their deposition through targeting the responsible host ligases, or possibly by wholesale inhibition of the host ubiquitination machinery. Other pathways targeted by ubiquitin-directed effectors include those regulating inflammation and autophagy [3,12]. Because the biological functions of many effectors remain incompletely understood, a pathway-based classification is currently not feasible for all proteins. The present review therefore uses molecular function as its primary organizing principle. A complete list of known effectors, their specificity, and their substrates is provided in Supplementary Table S1. The following sections survey the different classes of ubiquitin-directed effectors. The ‘Discussion’ section synthesizes these findings and considers the broader principles that emerge.

## Deubiquitinases

To date, more than 40 bacterial effectors have been reported to exhibit DUB activity, enabling them to remove ubiquitin marks placed by host ligases. DUBs are also common in eukaryotes, where they serve to regulate ubiquitin-dependent pathways [13]. Eight distinct classes of eukaryotic DUBs have been identified: one metalloprotease family (JAMM) and seven families of cysteine proteases (USP, UCH, OTU, Josephin, MINDY, ZUFSP, and VTD). DUB classes differ in their catalytic properties, with USP-type DUBs being largely substrate-specific, while OTU-type DUBs often target specific linkage types.

All known bacterial DUB effectors are cysteine proteases and adopt the papain fold (CA-clan in OTU, USP, VTD, and Josephin enzymes) or a circularly permutated version called the adenain fold (CE-clan) [14]. Most bacterial DUBs exhibit structural similarity to their eukaryotic counterparts, suggesting that they might have been acquired from host organisms. Typical intracellular Gram-negative bacteria encode one or two DUBs with little linkage specificity, sometimes showing a preference for K63-linked chains [15–17]. A few bacteria with a broad host range, like *Legionella pneumophila* and *Simkania negevensis*, possess a significantly expanded DUB repertoire [18,19].

### CE-clan

A large number of bacterial DUBs belong to the CE-clan, an enzyme superfamily that, in eukaryotes, cleaves ubiquitin-like modifiers such as NEDD8 or SUMO rather than ubiquitin itself [17,20]. Among bacterial effectors, CE-clan enzymes typically target ubiquitin, but some can also cleave SUMO chains or even catalyze lysine acetylation instead of acting as (iso-)peptidases. Among the first discovered bacterial DUBs were **ChlaDUB1** (Cdu1) and **ChlaDUB2** (Cdu2) from *Chlamydia trachomatis* [21]. Both enzymes cleave K63-linked ubiquitin chains and are anchored to the membranes of *Chlamydia*-containing vacuoles, known as ‘inclusions’. Thus, the ChlaDUBs are ideally situated to remove ubiquitin chains from the inclusion membranes. Notably, the anchoring of DUBs to the bacterial or BCV surface is a common strategy for such effectors. Besides their membrane-proximal activity, ChlaDUBs inhibit apoptosis and NF-κB activation [22,23]. Interestingly, ChlaDUB1 also exhibits acetyltransferase activity using the same active site [24]. Recently, **SnCE1** from the related bacterium *S. negevensis* was shown to cleave both SUMO and ubiquitin chains while also catalyzing lysine acetylation [19,25].

Among the enterobacteria, *S. enterica, Escherichia coli*, and *Shigella flexneri* encode related CE-clan DUBs. The *Salmonella* effector **SseL** primarily targets K63-linked ubiquitin chains and attaches peripherally to the BCV membrane through its N-terminal non-catalytic domain [20,26]. Various functionalities have been described, such as the inhibition of NF-κB activation and autophagy. The closely related effectors **ElaD** from *E. coli* and **ShiCE** from *S. flexneri* share both the linkage selectivity and the N-terminal membrane attachment domain but are less thoroughly characterized [20]. The single CE-clan enzyme **YopJ** from *Yersinia* was initially proposed to function as a deSUMOylase or a DUB [27,28] but was subsequently shown to be a serine/threonine acetyltransferase that inhibits host kinase signaling, similar to its *Salmonella* homolog **AvrA** [29]. Other bacteria encode DUBs with more diversified functionalities. *Legionella pneumophila* encodes seven proteins with CE-clan domains. In four of them (**SidE, SdeA, SdeB**, and **SdeC**), these domains catalyze K63-directed deubiquitination *in vitro* [30]. This activity may be connected to the unusual phosphoribosyl-linked ubiquitination (PR-Ub) catalyzed by the full-length proteins, which will be discussed in more detail below [31]. **LupA** uses its DUB activity to protect the unrelated *Legionella* effector LegC3 from ubiquitination [32]. Two further *Legionella* CE-clan proteins **Lem17** and **LegCE** lack DUB activity [20,33].

The proteobacterial plant pathogens *Xanthomonas campestris* and *Bradyrhizobium* encode the CE-clan effectors **XopD** and **NopD**, which possess dual deSUMOylase/DUB activity and disrupt transcriptional responses in their plant hosts [20,34,35]. An unusual DUB function is observed in the **CidB** protein of *Wolbachia* strains residing within the cytoplasm of insect cells [36]. This CE-type DUB acts as a sex-specific toxin and, together with the genetically linked antitoxin CidA, forms a gene drive system that mediates cytoplasmic incompatibility. Consequently, eggs from *Wolbachia*-free females are incompatible with sperm from *Wolbachia*-carrying males, thereby enhancing the vertical transmission of *Wolbachia* through the female germline. Other *Rickettsiales* also encode CE-clan DUBs: **OtDUB** from the scrub typhus pathogen *Orientia tsutsugamushi* cleaves longer ubiquitin chains with little linkage selectivity [37], while **RickCE** from *Rickettsia bellii* shows a preference for K63-linked chains [20].

### OTU

Several bacterial DUBs are related to the eukaryotic OTU family, with some exhibiting the characteristic linkage specificity of OTUs. Unlike the CE-clan DUB of *C. trachomatis*, other *Chlamydiae* such as *C. pneumoniae* and *C. caviae* encode a **ChlaOTU** [38]. The *Chlamydia*-related pathogen *S. negevensis* encodes **SnOTU**, a membrane-anchored DUB specific for linear chains [19]. This specificity is reminiscent of human OTULIN [39], although the enzymes belong to different OTU subfamilies. *Legionella pneumophila* contains four active OTU-type DUBs, named LotA-D (also known by their earlier names Lem21, Ceg23, Lem27, and Ceg7) [18,40–44]. **LotA/Lem21** is particularly noteworthy because it has two OTU domains: the N-terminal domain is specific for K6-linked chains, while the C-terminal domain favors long ubiquitin chains without linkage selectivity [40,45–47]. The role of K6 chains in the antibacterial defense remains uncertain. **LotB/Ceg23** and **LotC/Lem27** prefer K63 and K48 linkages, respectively, while **LotD/Ceg7** can cleave various chain types [43,44]. **SpAID** is a male-killing OTU-domain toxin originating from the insect endosymbiont *Spiroplasma poulsonii*, which is transmitted through the female germline [48]. Although SpAID's function is reminiscent of *Wolbachia* CidB, its DUB primarily serves to protect the protein itself from proteasomal degradation. Many additional bacterial OTU-DUBs have been identified recently in a large-scale survey, including **RickOTU** from *Rickettsia massiliae*, **BurkOTU** from *Burkholderia ambifaria*, **EschOTU** from *Escherichia albertii*, and **wPipOTU/wMelOTU** from endosymbiotic *Wolbachia* species that do not encode CidB-like DUBs [43].

### USP

In eukaryotes, the USP family represents the largest group of DUBs, yet only a few bacterial USP enzymes have been identified. The most thoroughly studied example is **TssM** from *Burkholderia pseudomallei*, the pathogen responsible for melioidosis [49]. TssM is a highly active DUB that cleaves K63, K48, and K11-linked chains. Despite its significant structural divergence from the eukaryotic USP consensus, it is unmistakably part of this family [50,51]. Its unique preference for ester-linked ubiquitin makes it an ideal candidate for removing RNF213-mediated LPS ubiquitination [51]. Three more conventional USP enzymes were identified in *S. negevensis*, among which **SnUSP1** and **SnUSP2** are highly active linkage-promiscuous DUBs, while **SnUSP3** shows minimal activity against ubiquitin chains [19].

### Josephins and clippases

Several bacteria encode DUBs that resemble the eukaryotic Josephin/Ataxin-3 family [52]. While some bacterial Josephins, like the K63-selective **PaJOS** from *Pigmentiphaga aceris*, function as conventional DUBs, most other members exhibit an unconventional ‘clippase’ activity. In this reaction, the enzyme cleaves after Arg-74 within the ubiquitin C-terminus, rendering ubiquitin removal irreversible. This is because the C-terminally shortened ubiquitin cannot be activated, and the diGly remnant left on the substrate lysine prevents re-modification [52]. Similarly, unconventional cleavage modes had previously been observed for two UBL-proteases: **RavZ**, a CE-clan effector from *L. pneumophila*, delipidates Atg8 proteins irreversibly by cutting before the C-terminal glycine residue [53], while Lbpro from foot and mouth disease virus cleaves ISG15 before the C-terminal di-glycine [54]. The latter enzyme could be engineered to also recognize ubiquitin [55], albeit with modest specific activity. Among the bacterial ubiquitin-clippases, **BpJOS** from *Burkholderia pyrrocinia* and **BcJOS** from *Burkholderia catarinensis* demonstrate particularly high and pan-specific clippase activity, whereas **SnJOS1/2** from *S. negevensis* are less active. **PcJOS** from *Parachlamydia sp*. is a highly active M1-specific clippase. Mass spectrometry-based detection of clippase-generated diGly(K) remnants makes these enzymes valuable tools for studying the ubiquitin system, as exemplified by the recent application of BpJOS to discover glycogen-ubiquitination [56].

### Other bacterial DUBs

The recently described VTD DUB family also comprises bacterial members [57]. **SnVTD** from *S. negevensis* and **WcVTD1** from *Waddlia chondrophila* both catalyze K6-specific deubiquitination, while the more divergent **WcVTD2** is K63-specific. Thus, the *Chlamydia*-like pathogens *Simkania* and *Waddlia* share with *L. pneumophila* the capability to target K6-linked chains, although the reasoning behind this activity remains unclear. Similar to *Simkania, L. pneumophila* encodes a linear-specific DUB known as **RavD**, which is unrelated to the M1-specific SnOTU or any other DUB [58]. RavD attaches to BCVs through its C-terminal domain, preventing the endolysosomal maturation of these vacuoles and interfering with ubiquitin-mediated NF-κB activation. It appears that these two unrelated bacteria have independently evolved linear-specific DUBs, suggesting that M1-linked ubiquitin represents an important host target for distinct intracellular bacteria. Another DUB without affiliation to eukaryotic DUB classes is **EmcB** from the Q-fever pathogen *Coxiella burnetii* [59]. This enzyme cleaves multiple ubiquitin linkages and has been shown to block the Rig-I-induced interferon response.

## Ligases

Both intracellular bacteria and those primarily living extracellularly can secrete ubiquitin ligase effectors into host cells. In most well-studied instances, these ligase effectors function to target host proteins for proteasomal degradation, thereby modifying cellular signaling to the bacterium's advantage [3,60,61]. Only a subset of bacterial ligases is homologous to host enzymes: eukaryotic E3 classes that facilitate ubiquitin transfer from E2 to the substrate, such as RING- and Ubox-proteins, are also present in bacteria. However, bacterial counterparts of the eukaryotic transthiolating ligase classes (HECT, RBR, RCR, and RZ) have not yet been identified. Instead, bacteria have developed distinct transthiolating E3 classes, which are prevalent in human pathogens and target key proteins involved in antibacterial defense.

### Bacterial HECT-like ligases

**SopA** from *S. enterica* and **NleL** from enterohemorrhagic *E. coli* (EHEC) are the founding members of a family of transthiolating ligases that mimic the mechanism of HECT ligases without being related to them [62,63]. Recently, the family was expanded by characterizing the putative effectors **PvHECT** from *Proteus vulgaris*, **PsHECT** from *Pantoea stewartii*, **EaHECT** from *Erwinia amylovora*, and **VsHECT** from *Verrucomicrobia spp* [64]. The catalytic domain is located at the C-terminus and comprises two flexibly linked lobes [64]. The N-lobe binds the loaded E2 and transfers ubiquitin to a cysteine residue in the C-lobe, from where it is transferred onto the substrate. The N-terminal non-catalytic region of these proteins consists of β-strand repeats that recognize the substrates. SopA targets the host ligases TRIM56 and TRIM65, both of which are involved in innate immunity, while NleL targets caspase-4 and ROCK1/2 kinases, thereby inhibiting inflammatory cell extrusion and pyroptosis [65,66].

### NEL ligases

A second, considerably larger family of bacterial transthiolating ligases was identified later and named NEL (novel E3 ligase) [67,68]. Typical NEL ligases are characterized by an N-terminal substrate-binding leucine-rich repeat region, followed by a catalytic NEL domain that recognizes the charged E2 and contains the catalytic cysteine, yet lacks a HECT-like bi-lobal structure. NEL ligases are prevalent in *Salmonella* and *Shigella* but are also found in other proteobacterial pathogens of animals and plants. All well-studied examples modify their targets primarily through K48 chains. In *S. enterica*, **SspH1** targets the PKN1 kinase, while the closely related **SspH2** targets NOD1, a cytoplasmic sensor for bacterial proteoglycans [68,69]. The third *Salmonella* NEL ligase, **SlrP**, targets host thioredoxin and the ER-associated chaperone DNAJB11 [70]. *Shigella flexneri* contains up to eleven NEL ligases, with some being part of the core genome and others plasmid-encoded, thus not present in every strain [68,71]. **IpaH1.4** and **IpaH2.5** inhibit NF-κB activation by targeting the ligase HOIP, while **IpaH1.4** additionally targets the LPS-directed ligase RNF213 [72–75]. **IpaH7.8** prevents epithelial pyroptosis by targeting the pyroptotic pore-forming gasdermins GSDMD and GSDMB; it also ubiquitinates glomulin (GLMN), a regulator of Cullin-based ubiquitin ligases [76–78]. **IpaH4.5** targets the proteasomal ubiquitin receptor RPN13, as well as several innate immunity regulators such as TBK1 and NLRP3 [79–81]. Similarly, **IpaH9.8** modulates immunity signaling by ubiquitinating NEMO and several members of the guanylate-binding protein (GBP) family [82–84]. The NEL ligases **YspE1** and **YspE2** from *Yersinia pestis* also target GBP-based defenses [85]. The *Ralstonia solanacearum* effectors **RipAR** and **RipAW** inhibit pattern-triggered immunity in plants, potentially by ubiquitinating an immune receptor complex composed of FLS2, XLG2, and BIK1 [86,87].

### SNL ligases

The effectors **SidC, SdcA**, and **SdcB** constitute a small, *Legionella*-specific family of transthiolating ligases [88,89]. Although they are not related to HECT-like or NEL ligases, crystal structures complexed with ubiquitin-charged E2s reveal a conserved and catalytically essential cysteine residue adjacent to the catalytic E2-cysteine [90]. The N-terminal domain, which houses the catalytic cysteine and E2 binding site, is referred to as the SNL (SidC N-terminal ligase) domain. Positioned C-terminally to the SNL domain is a lipid-binding domain that facilitates localization at the *Legionella*-containing vacuole membrane. Among the substrates of SidC and SdcA are small Rab-GTPases and SNARE proteins, which are key regulators of vesicular traffic [88].

### RING and UBox ligases

Several bacteria have added eukaryotic RING or UBox domains to their effector repertoire, two E3 types that work by allosterically activating E2s and bringing them into contact with substrates. Typical RING domains coordinate two Zn^2+^ ions for structural stabilization, while UBoxes stabilize the same fold without Zn coordination. The UBox protein **AvrPtoB** from *Pseudomonas syringae*, also known as **HopAB1/2/3**, suppresses plant antibacterial defenses and cell death by targeting the host kinase Fen and other proteins [91,92]. *Escherichia coli*-derived **NleG** forms a large family of bacterial Ubox-ligases with more than 10 copies in some EHEC strains and multiple members in other enterobacteria, including *S. enterica* and *Shigella sonnei* [93]. Only for a few NleG proteins’ information about ubiquitination targets is available. **NleG5-1** ubiquitinates nuclear MED15, a component of the mediator complex, while cytosolic **NleG2-3** targets the vesicular trafficking protein SNAP29 and hexokinase-2 [94]. *Salmonella* **StoD** is a structurally characterized member of the NleG family and preferentially elongates pre-existing ubiquitin chains [95]. A related Ubox family is formed by **GobX** from *L. pneumophila* and other *Legionellaceae*. It is localized to the Golgi apparatus through palmitoylation; specific ubiquitination targets have not yet been identified [96]. **LubX**, another *Legionella* effector, is better characterized and features two Ubox domains that closely resemble those of eukaryotic ligases [97]. The first Ubox appears to bind E2s, while the second Ubox recognizes the substrate SidH, an unrelated *Legionella* effector marked for ubiquitination and subsequent proteasomal degradation [98]. Consequently, LubX is regarded as a meta-effector, as it regulates the fate of another effector. A very divergent member of the Ubox family is found in the *Legionella* effector **RavN**, which lacks detectable sequence similarity to RING/Ubox ligases but shows a Ubox-related structure with a conserved E2-binding interface [99]. The latest addition to this list is **SnRING** from *S. negevensis* [100]. Unlike the previously mentioned proteins, SnRING coordinates Zn^2+^ ions through a somewhat atypical RING finger. While physiological substrates have yet to be identified, the protein interacts with several proteins located in the mitochondria and ER and can ubiquitinate some of them *in vitro*.

### CRL adaptors

In eukaryotes, Cullin–RING ligases (CRLs) are a major family of E3 ubiquitin ligases that use interchangeable substrate adaptor proteins to confer target specificity. These adaptors include F-box proteins, which assemble with Skp1 and Cullin-1 to form SCF ubiquitin ligases [101]. Several bacterial effectors have acquired F-box domains that allow them to hijack the cellular SCF system. Multiple effectors of *L. pneumophila* exhibit sequence similarity to the F-box consensus, with two of them demonstrated to be active. **AnkB**, an effector containing ankyrin repeats, targets the host actin-organizer parvin-B, thereby interfering with host ligases that target the same protein [102]. **LegU1** has also been shown to form active CRL complexes that target the host chaperone BAT3 [103]. **LicA**, another F-box protein from *Legionella*, binds to the host F-box partner Skp1 but does not seem to form an active ligase complex, while other predicted *Legionella* F-box proteins do not associate with host complexes at all [103]. Beyond *Legionella*, **VirF** is a functional F-box effector from *Agrobacterium tumefaciens*, which helps this bacterium to transform plant cells. Besides targeting the host transcription factor VFP4 for degradation, VirF also acts as a meta-effector targeting the bacterial VirE2 protein [104,105]. The plant pathogen *R. solanacearum* encodes seven related F-box proteins with additional leucine-rich repeats, collectively known as the **GALA** or **RipG** family [106]. Generally, the GALA proteins are essential for *Ralstonia*'s virulence across various host plants, although some redundancy and species-specificity have been noted. Several GALA/RipG proteins seem to target host chloroplast proteins, and specifically, **RipG6** has been reported to act against the tomato kinase RLCK-VIII-6 [107,108].

### Other ligases

Several bacterial effectors that do not belong to one of the established E3 families have been described as ubiquitin ligases, with varying degrees of biochemical and structural characterization. The effector **XopL** from *X. campestris* was found to have E3 activity attributed to a unique and cysteine-less domain termed ‘XL-box’ [109]. Among the substrates of XopL is the plant autophagy component SH3P2 [110]. *Legionella pneumophila* has been described to encode two more enigmatic E3 effectors without a known E2 binding site or mode of action. **Lug14** targets the host ubiquitin ligase ARIH2, causing increased inflammasome activation in macrophages [111], while **Lug15** targets the vesicular trafficking protein Sec 22b [112]. A particularly unusual E3 was recently identified in *Pseudomonas aeruginosa*: The secreted **Pul-1** protein is related to short-chain acyl-CoA dehydrogenases (SCAD) but lacks this enzymatic activity and rather catalyzes ubiquitin ligation via a non-conserved cysteine remote from the SCAD active site [113]. Despite these unexpected characteristics, Pul-1 shows robust ubiquitination activity *in vitro* and plays a role in regulating bacterial virulence. Additional highly unusual bacterial E3 candidates have been published, for which the mode of action remains unclear and the observed activity is weak or possibly indirect, such as **PM19_00185** from *Phytoplasma malii* and **TRP120** from *Ehrlichia chaffeensis* [114,115].

## Ubiquitin inhibitors and cross-linkers

Deubiquitination is not the only method to counter ubiquitin-based host defenses. Some bacteria use effectors that alter ubiquitin or key components of the ubiquitination pathway, modulating the host ubiquitin system more fundamentally. The cycle inhibiting factor (**CIF**) is a bacterial effector from pathogenic *E. coli* that inhibits the host cell cycle. Homologs, such as **CHBP** from *B. pseudomallei*, exist in other proteobacteria. These proteins are related to papain-fold cysteine proteases but act as glutamine deamidases targeting Gln-40 of ubiquitin and NEDD8 [116,117]. The resulting irreversible Q40E modification disrupts the E2-E3 transfer of ubiquitin, inhibiting host ubiquitination [118,119]. *Legionella pneumophila* encodes two CIF-related effectors, **MavC** and **MvcA**, which are capable of deamidating ubiquitin at Q40 [120]. However, **MavC** not only hydrolyzes Q40 but also crosslinks it to K92 and K94 of UBE2N, the K63-specific E2 enzyme [121–123]. Consequently, the host cell's ability to generate K63 chains is impaired. A similar effect is caused by the *Shigella* effector **OspI**, which is not related to CIF but also has deamidase activity. OspI targets UBE2N directly and deamidates Q100 to glutamate, thereby weakening the recognition by RING ligases [124,125]. **OspG**, another effector of *S. flexneri*, is an unusual protein kinase that binds to and inhibits Ubiquitin-E2 complexes [126,127] while simultaneously activating its own kinase activity, causing the phosphorylation of several substrates such as CAND1, a crucial regulator of Cullin-RING ligases [127,128]. A different modification on ubiquitin is introduced by **CteC**, an effector from *Chromobacterium violaceum*, which transfers ADP-ribose onto Thr-66 of ubiquitin, thereby inhibiting its transfer from E1 to E2 and thus cellular ubiquitination pathways [129].

### PR-ubiquitination

ADP-ribosylation is also at the center of a sophisticated ubiquitin-crosslinking system established by the *L. pneumophila* effector **SidE** and its paralogs **SdeA, SdeB**, and **SdeC** [130,131]. In a first reaction step, these enzymes transfer ADP-ribose onto Arg-42 of ubiquitin. Subsequently, a phosphodiesterase activity of the same protein cleaves the ADPR-ubiquitin, either releasing the hydrolysis products AMP and PR-Ub or simultaneously transferring the PR-Ub onto a substrate serine residue. Via this crosslink, the substrate becomes covalently attached to ubiquitin independent of its C-terminus. This so-called PR-Ub reaction requires neither ATP nor E1 and E2 enzymes; the energy is provided by NAD hydrolysis. Among the known substrates are many Rab-type GTPases such as Rab33b and Rab1, as well as ER/Golgi-associated proteins and autophagy factors [132]. While not sensitive to conventional DUBs, PR-Ubiquitination can be reversed through *Legionella*-encoded phosphodiesterases [132]. **DupA** and **DupB** are able to unlink PR-ubiquitinated substrates, yielding the unmodified substrate and PR-Ub. Through the combined action of **LnaB** (which re-attaches AMP to PR-ubiquitin) and **MavL** (which removes the resulting ADPR group), ubiquitin can be restored as well [133–135].

## Is there a unifying big picture?

When considering the complete repertoire of bacterial ubiquitin effectors, it becomes apparent that they do not represent a functionally uniform group with a single overarching purpose. Rather, these effectors constitute a diverse collection of molecular tools that have been independently adapted by different bacterial species to manipulate host ubiquitin systems in distinct ways. Viewing this repertoire from a bacteria-centric perspective reveals a complex picture in which ubiquitin modification serves multiple, sometimes seemingly opposing, roles during the different stages of infection.

One prominent theme is the direct remodeling of ubiquitin deposition at the bacterial surface or on BCVs (Figure 1A). Bacterial effectors restrict ubiquitin accumulation on these pathogen-associated membranes through several complementary mechanisms. Surface-associated DUBs directly remove ubiquitin marks, whereas bacterial ubiquitin ligases may counteract host ubiquitination indirectly by targeting the host E3 ligases responsible for their deposition. Other effectors that broadly perturb host ubiquitin pathways may further contribute to limiting ubiquitination at the pathogen–host interface. A well-defined strategy is employed by BCV-associated DUBs, including *Legionella* LotA, RavD, and LotB, *Salmonella* SseL, and *C. trachomatis* Cdu1, which are recruited or anchored to pathogen-containing vacuoles through dedicated lipid-targeting or transmembrane domains [23,26,40,42,136]. Other DUBs may function similarly; for example, the *S. negevensis* DUBs SnVTD and SnOTU contain predicted transmembrane domains, consistent with a localization at the inclusion membrane [19]. Among free-living cytosolic bacteria, the *B. pseudomallei* DUB TssM employs an analogous strategy by removing ubiquitin from the bacterial surface through cleavage of the ester linkage between lipopolysaccharide and ubiquitin [51]. Bacterial ubiquitin ligase effectors can also prevent the accumulation of bacteria-associated ubiquitin. The *Shigella* NEL ligase IpaH1.4 promotes the degradation of RNF213, which initiates bacterial LPS ubiquitination, and of the linear ubiquitin chain assembly complex (LUBAC), which amplifies this signal by assembling M1-linked ubiquitin chains. Thus, IpaH1.4 antagonizes both the initiation and amplification of antibacterial ubiquitination [73–75,137].

**Figure 1:**
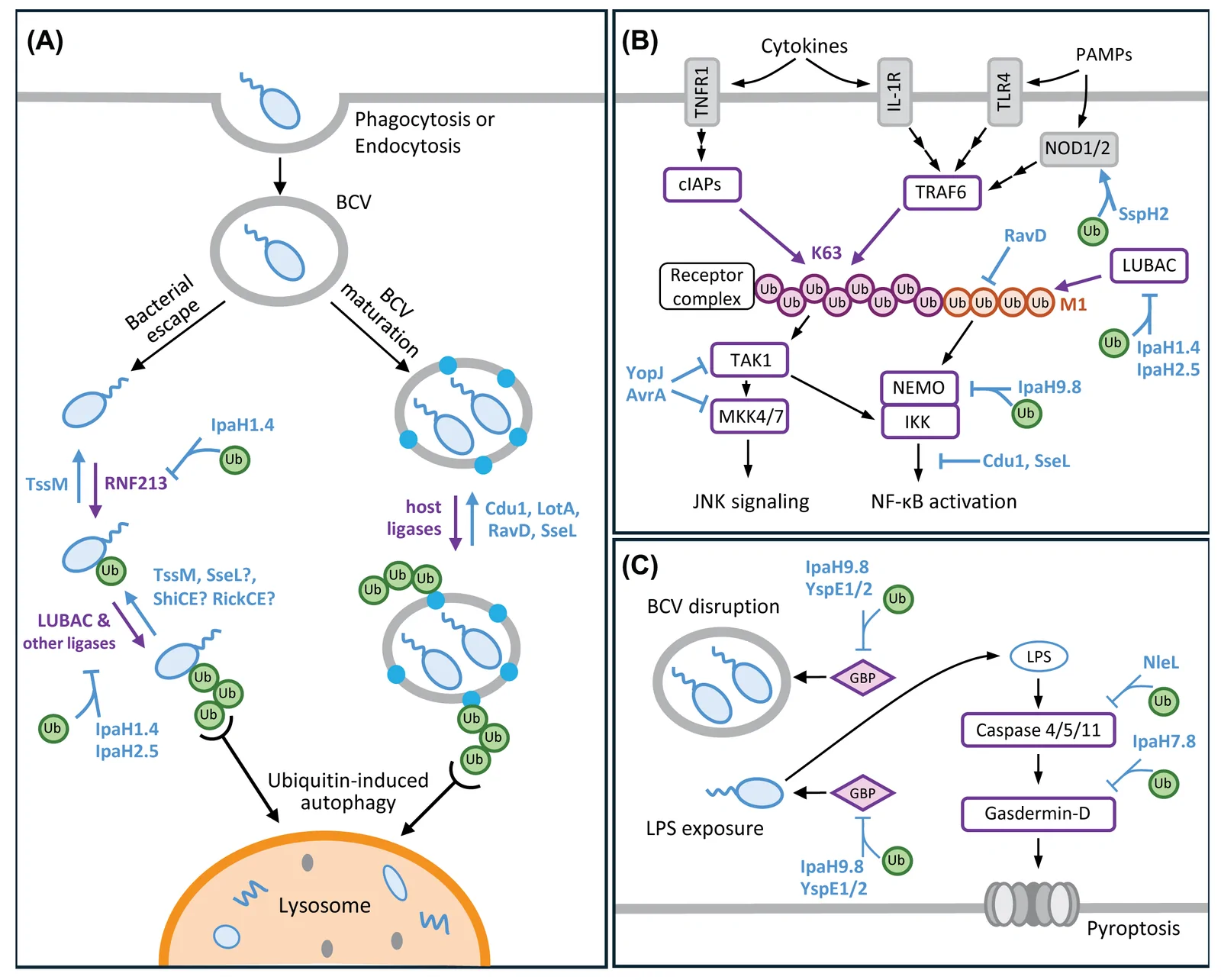
Selected areas of ubiquitin-directed effector activity Bacterial effectors and their activities are shown with light blue letters/arrows, while host components and activities are shown in purple. Ubiquitin is generally shown as green circles, unless present in K63 chains (purple) or M1 chains (red). (**A**) Effectors active at the surface of bacteria (left branch) or BCVs (right branch) are shown. Host ligases (purple) attach ubiquitin marks to the bacterial or BCV surface, which can be reversed by bacterial DUB effectors (blue). In addition, the host ligases themselves get targeted for degradation by bacterial ligase effectors (shown associated with green ubiquitin circles). Remaining ubiquitin tags can target bacteria and BCVs for lysosomal degradation (bottom). (**B**) Effectors working in NF-κB signaling. Pathways initiated by PAMP- and cytokine receptors converge on receptor-associated K63 chains (purple circles), which can be capped by LUBAC-installed M1 chains (red). These chains serve as activation platforms for NF-κB and JNK signaling. Bacterial ligase effectors (light blue) target several components, mostly inhibiting the NF-κB pathway, with the exception of the activating ubiquitination by SspH2. (**C**) Effectors working in LPS-signaling/pyroptosis. Host GBP proteins (purple diamonds) can attack BCVs and LPS-containing bacterial membranes, causing LPS exposure and activation of the Caspase-4/5/11-mediated pyroptosis pathway. Bacterial E3 effectors (light blue) target various components of the pathway for degradation, thus preventing LPS-induced pyroptosis.

A second major functional category comprises effectors that target the **NF-κB signaling** pathway [138] (Figure 1B). Bacterial components are detected either extracellularly, by the Toll-like receptor TLR4, or in the cytoplasm, by the NOD1/2 proteins; both routes converge on activation of TRAF6, a RING-type E3 ubiquitin ligase that installs K63-linked ubiquitin chains on itself and on other components of the receptor−proximal complex. Comparable K63-chain-generating steps occur downstream of pro-inflammatory cytokine receptors such as TNFR1 and IL-1R. Receptor-associated K63 chains act as a signaling scaffold, recruiting the TAK1-TAB 2/3 complex; the kinase activity of TAK1 then both activates the IκB kinase (IKK) complex, triggering IκB degradation and NF-κB activation, and drives JNK signaling through the MKK4/7 axis. Signaling through the IKK complex is reinforced by the LUBAC ligase complex, which caps the K63 chains with linear chains and/or directly modifies the IKK subunit NEMO with M1 chains; NEMO in turn docks onto this linear ubiquitin, stabilizing the complex and further boosting IKK activation. As a master regulator of innate immunity, NF-κB is an attractive target for bacterial effectors. Receptor-associated linear ubiquitin chains can be directly removed by the M1-specific *Legionella* DUB RavD, or their generation can be prevented by targeting LUBAC, as demonstrated for the *Shigella* E3 ligases IpaH1.4 and IpaH2.5, which promote degradation of the LUBAC subunit HOIP. Another *Shigella* E3, IpaH9.8, targets the IKK component NEMO for ubiquitination and proteasomal degradation, while the DUBs Cdu1 from *Chlamydia* and SseL from *Salmonella* inhibit NF-κB activation by preventing ubiquitination and subsequent degradation of IκBα. The JNK branch of the pathway is targeted by *Yersinia* YopJ and *Salmonella* AvrA, two CE-clan acetyltransferases that inhibit MAPK signaling by acetylating critical Ser/Thr residues in the activation loops of their kinase targets [139].

A third major functional category includes ubiquitin-directed effectors that modulate **LPS-dependent signaling** and cell death (Figure 1C). GBPs recognize and remodel the LPS-containing membranes of cytosolic Gram-negative bacteria, thereby promoting exposure of lipid A and recruitment and activation of human caspase-4/5 or murine caspase-11 [140,141]. GBPs can also target BCVs and contribute to their membrane disruption, thereby promoting bacterial access to the cytosol [142,143]. Cytosolic LPS directly activates caspase-4/5/11, initiating the non-canonical inflammasome pathway. These caspases cleave gasdermin D (GSDMD), whose N-terminal fragment forms plasma-membrane pores, causing pyroptotic cell death [144]. Bacterial effectors can interfere with this pathway at multiple levels. The *Shigella* ligase IpaH9.8 and the *Yersinia* ligases YspE1/2 all target GBPs for K48-linked ubiquitination and proteasomal degradation. *Shigella* IpaH7.8 acts further downstream and targets human GSDMD and GSDMB, thereby suppressing pyroptosis [77,78]. The *E. coli* ligase NleL targets the LPS-sensing CARDs of caspase-4 and caspase-5 for ubiquitination, thereby inhibiting non-canonical inflammasome activation [66].

Inflammation is not universally detrimental to bacterial pathogens. Depending on their lifestyle and stage of infection, some bacteria can benefit from inflammatory responses. Consistent with this notion, IpaH7.8 can also promote inflammasome activation by targeting the inflammasome regulator GLMN for proteasomal degradation, thereby inducing pyroptotic cell death of infected macrophages [76]. The apparently opposing effects of IpaH7.8 on pyroptosis can be explained, at least in part, by the distinct requirements of *Shigella* in different host cell types: in macrophages, pyroptosis promotes bacterial escape and dissemination, whereas in epithelial cells, premature pyroptosis appears to be detrimental [76,77]. In *Salmonella* infections, the HECT-like E3 ligase SopA contributes to intestinal inflammation by targeting mammalian TRIM ligases [62,65], while the NEL ligase SspH2 modifies mammalian NOD receptors and enhances inflammatory signaling [69]. Together, these examples illustrate that bacterial ubiquitin-directed effectors do not simply suppress host immunity but rather fine-tune inflammatory responses to increase pathogen fitness in a given host environment.

On the other hand, not all cellular changes induced by bacterial effectors are beneficial for the pathogen. In plants, effector-triggered immunity (ETI) represents an important defense mechanism in which immune receptors detect pathogen effectors or the cellular changes caused by their activity [145]. Although the term ETI is rarely used for animal immunity, similar mechanisms may exist, as illustrated by *Shigella* IpaH7.8, whose ubiquitination of the inflammasome sensor NLRP1B triggers inflammasome activation through ‘functional degradation’ [146].

Two classes of ubiquitin-directed effectors do not readily fit into the functional categories described above. One comprises so-called meta-effectors, which regulate the stability or ubiquitination status of other bacterial effectors [97]. Examples include *Legionella* LupA, which deubiquitinates LegC, as well as the ubiquitin ligases LubX and *Agrobacterium* VirF, which promote the proteasomal degradation of SidH and VirE, respectively. These proteins primarily function as regulators of bacterial effector activity rather than directly manipulating host ubiquitin signaling. A second class consists of effectors that broadly interfere with the host ubiquitin machinery without obviously targeting a specific cellular pathway. Examples include the deamidases Cif, CHBP, and MavC, which modify ubiquitin or NEDD8; the ubiquitin ADP-ribosyltransferase CteC; the UBE2N-directed deamidase OspI; and clippases, which truncate ubiquitin at its C-terminus. These activities are thought to inhibit ubiquitination globally or selectively impair K63-linked ubiquitin signaling. However, the precise advantage of such broad inhibition for bacterial fitness remains incompletely understood.

More generally, several intracellular pathogens encode remarkably large repertoires of ubiquitin-directed effectors, raising the question of how these enzymes avoid interfering with one another. This issue is particularly evident for pathogens such as *Legionella*, which deploy numerous ubiquitin ligases, DUBs, and ubiquitin-modifying enzymes simultaneously. Several mechanisms may contribute to their functional compatibility. Spatial segregation can confine individual activities to BCVs or distinct host compartments, as exemplified by membrane-associated DUBs. Enzymatic specialization may provide another layer of separation, with different effectors exhibiting distinct preferences for ubiquitin chain linkages, substrates, or modified forms of ubiquitin. Finally, some bacterial ubiquitin ligases may have evolved to utilize effector-modified ubiquitin variants that are poor substrates for the host ubiquitination machinery, thereby remaining active even when host ubiquitination has been broadly inhibited. The phosphoribosyl ubiquitination pathway of *Legionella* provides an example of such orthogonal chemistry [130]. Determining how large bacterial effector repertoires are coordinated in time and space and how they avoid mutual antagonism remains an important challenge for future studies.

## Perspectives

- Intracellular bacteria, residing either in membrane-enclosed vacuoles or escaping into the cytosol, secrete ubiquitin-directed effectors into the host cell to aid in evading ubiquitin-based host defenses.
- Bacterial effectors include ubiquitin writers, erasers, and modulators, which can prevent bacterial recognition, disrupt host signal transduction, or prevent and reverse ubiquitin tagging.
- In addition to conventional deubiquitinases and ubiquitin ligases acquired from eukaryotic hosts, new effectors with unconventional activities are being discovered, some of which serve as valuable tools for studying the ubiquitin system independently of an infection scenario.

## Supplementary Material

Supplementary Table S1

## Open Access

Open access for this article is provided via our Subscribe to Open programme, made possible through the support of our subscribing institutions. Learn more here: https://portlandpress.com/pages/open_access_options_and_prices#conditional.

## Abbreviations

ADPR: ADP-ribose
BCVs: bacteria-containing vacuoles
CIF: cycle-inhibiting factor
CRL: Cullin-RING ligase
DUB: deubiquitinase
E1: ubiquitin activating enzyme
E2: ubiquitin conjugating enzyme
E3: ubiquitin ligase
EHEC: enterohemorrhagic *E. coli*
ETI: effector-triggered immunity
FMDV: foot and mouth disease virus
GBP: guanylate-binding protein
GLMN: glomulin
GSDMD: gasdermin D
HECT: homologous to E6AP C-terminus
IKK: IκB kinase
LPS: lipopolysaccharide
LUBAC: linear ubiquitin chain assembly complex
NAD: Nicotinamide Adenine Dinucleotide
NEL: novel E3 ligase
PAMP: Pathogen-associated molecular pattern
PR-Ub: phosphoribosyl-linked ubiquitin
SCAD: short-chain acyl-CoA dehydrogenases
SNL: SidC N-terminal ligase
SCF: Skp1–Cullin–F-box complex
UBL: Ubiquitin-like protein
