# Supplementary material for: Bacterial effectors targeting the host ubiquitin system
Source: Biochem Soc Trans. 2026 Oct 29;54(11):1553–67. doi: 10.1042/BST20260089 (PMC13720258; doi:10.1042/BST20260089)
Supplement: Supplementary file 1 [file BST-2026-0089C_supp.pdf]

Supplementary Table 1: Information on currently known ubiquitin-directed effectors.

All effector classes (DUBs, E3, others) are combined in a single table to allow grouping/filtering by bacterial species, bacterial class or intracellular life style. DUBs and DUb-like enzymes are shown in blue, E3-ligases and ligase-components are shown in green, other effectors are shown in yellow.

Explanation of fields and abbreviations used therein:

Field Name	contents	Explanation of abbreviations etc.
**Effector name and synonyms**	most common names, same as in text	
**Effector family**	Classification by sequence relationship	**CE-clan, OTU, USP, Josephin, VTD**: names of DUB families, explained in text. **bHECT, NEL, SNL, Ubox, RING, F-box**: names of E3 families, explained in text. **Cif** : Q40 deamidase family. **ART**: member of the ADP-ribosyltransferase family. **Kinase**: member of the protein-kinase family. **SidE**: member of the Legionella SidE family, consisting of multiple domains. **AMPylase**: member of the AMP-transferase family. **Macro**: Member of the macro-domain ADP-ribosyl hydrolase family.
**Molecular function**	brief description of enzymatic function(s). Multiple functions possible	**DUB**: deubiquitinase. **UBL-protease**: active on other modifiers (SUMO, NEDD8). **AT**: Acetyltransferase. **Clippase**: protease with destructive cleavage mode (before C-terminal glycine(s). **E3**: ubiquitin ligase enzyme. **E3-adaptor**: part of a Cullin-RING ligase complex. **Q40**: Deamidases/Crosslinkers acting on Gln-40 of ubiquitin or Nedd8. **MARylation**: mono ADP-ribosylation. **PR**: phosphoribosyl.
**Modifier specificity**	Modifier(s) targeted by effector	
**Source bacterium**	binomial species name. If effector is present in multiple related bacteria, the best-characterized species is given	
**bacterial lifestyle**	Where to the bacteria typically replicate	**BCV**: within bacteria-containing vacuoles. **cytoplasmic**: directly within the cytoplasm. **Extracellular**: mostly outside of host cells.
**bacterial class**	Taxonomic class of the bacterial species	
**main hosts**	indication of typical host range. For effectors existing in multiple related species, the host range of the best-studied effector-encoding species is given	
**linkage specificity**	When applicable, the ubiquitin chain specificity of the effector is indicated	**promiscous**: effector shows no clear linkage preference. D**egradative chains** indicates E3 enzymes for which chain specificity has not been formally determined, but are assumed to be K48/K11 as they are reported to cause proteasomal degradation
**prominent substrates**	List of known substrates (given as gene names) with literature support. See text for references.	
**comments**	Additional information, if available	
**Uniprot-URL**	Clickable link to the Uniprot database entry of the effector sequence	

#	Effector name and synonyms	Effector family	Molecular function	Modifier specificity	Source bacterium	bacterial lifestyle	bacterial class	main hosts	linkage specificity	prominent substrates	comments	Uniprot-URL
1	Cdu1 / ChlaDUB1	CE-clan	DUB, AT	Ubiquitin (some Nedd8)	Chlamydia trachomatis	BCV	Chlamydiia	humans	K63>>K48,K11	MCL-1, GLUT	transmembrane domain	O84876
2	Cdu2 / ChlaDUB2	CE-clan	DUB	Ubiquitin	Chlamydia trachomatis	BCV	Chlamydiia	humans	mostly mono	unknown	transmembrane domain	O84875
3	SnCE1	CE-clan	DUB, UBL-protease, AT	Ubiquitin, SUMO	Simkania negevensis	BCV	Chlamydiia	amoeba, humans	K63, K11, K48	unknown	related to Cdu1	F8L4W9
4	SseL	CE-clan	DUB	Ubiquitin	Salmonella enterica	BCV + cytoplasmic	gammaproteo-bacteria	animals	K63>>K48,K11	BCV surface, S100A6, hnRNP-K,	PI-lipid binding domain	Q8ZNG2
5	ElaD	CE-clan	DUB	Ubiquitin	Escherichia coli	extracellular	gammaproteo-bacteria	vertebrates	K63>>K48,K11	unknown	PI-lipid binding domain	Q47013
6	ShiCE	CE-clan	DUB	Ubiquitin	Shigella flexneri	cytoplasmic	gammaproteo-bacteria	humans	K63>>K48,K11	unknown	PI-lipid binding domain	F5N5C0
7	YopJ	CE-clan	AT	none	Yersinia spp.	extracellular	gammaproteo-bacteria	vertebrates	no DUB	signaling kinases (IKK, TAK, MKKs)	pure acetyltransferase	O68718
8	AvrA	CE-clan	AT	none	Salmonella enterica	BCV + cytoplasmic	gammaproteo-bacteria	animals	no DUB	signaling kinases (Jnk pathway)	pure acetyltransferase	E8XKZ3
9	SidE	CE-clan	DUB	Ubiquitin	Legionella pneumophila	BCV	gammaproteo-bacteria	amoeba, humans	K63>K48,K11	BCV-surface	Also PR-ligase domains	Q5ZYX7
10	SdeA	CE-clan	DUB	Ubiquitin (some Nedd8)	Legionella pneumophila	BCV	gammaproteo-bacteria	amoeba, humans	K63>K48,K11	BCV-surface	Also PR-ligase domains	Q5ZTK4
11	SdeB	CE-clan	DUB	Ubiquitin	Legionella pneumophila	BCV	gammaproteo-bacteria	amoeba, humans	K63>K48,K11	BCV-surface	Also PR-ligase domains	Q5ZTK5
12	SdeC	CE-clan	DUB	Ubiquitin	Legionella pneumophila	BCV	gammaproteo-bacteria	amoeba, humans	K63>K48,K11	BCV-surface	Also PR-ligase domains	Q5ZTK8
13	LupA	CE-clan	DUB	Ubiquitin	Legionella pneumophila	BCV	gammaproteo-bacteria	amoeba, humans	promiscuous	Legionella LegC3	meta-effector, transmembrane domain	Q5ZWD7
14	Lem17	CE-clan	AT	none	Legionella pneumophila	BCV	gammaproteo-bacteria	amoeba, humans	no DUB	JAK1 kinase	pure acetyltransferase	A0AAN5PJ36
15	LegCE	CE-clan	AT	none	Legionella pneumophila	BCV	gammaproteo-bacteria	amoeba, humans	no DUB	unknown	pure acetyltransferase	Q5ZRH5
16	XopD	CE-clan	DUB, UBL-protease	Ubiquitin, SUMO, (some Nedd8)	Xanthomonas campestris	extracellular	gammaproteo-bacteria	plants	K48>others	plant SIERF4 (deSUMOylation)	mainly characterized as deSUMOylase	Q8RJQ0
17	NopD	CE-clan	DUB, UBL-protease	Ubiquitin, SUMO	Bradyrhizobium spp.	BCV	alphaproteo-bacteria	plants	K48>others	nodulation pathway		A0A2M8R782
18	CidB	CE-clan	DUB	Ubiquitin	Wolbachia	BCV	alphaproteo-bacteria	insects	K63>others	nuclear import factors		Q73HD4
19	OtDUB	CE-clan	DUB	Ubiquitin	Orientia tsutsugamushi	cytoplasmic	alphaproteo-bacteria	mammals	K33>others	unknown	prefers long chains	A0A2U3QWA1
20	RavZ	CE-clan	UBL-clippase	ATG8	Legionella pneumophila	BCV	gammaproteo-bacteria	amoeba, humans	no DUB	lipidated Atg8	cleaves lipidated LC3 before C-terminal Gly	Q5ZUV9
21	RickCE	CE-clan	DUB	Ubiquitin (some Nedd8)	Rickettsia bellii	cytoplasmic	alphaproteo-bacteria	arthropods	K63>K11	unknown		Q1RK35
22	ChlaOTU	OTU	DUB	Ubiquitin	Chlamydia pneumoniae	BCV	Chlamydiia	vertebrates	unknown/weak	NDP52	transmembrane domain	Q9Z868
23	SnOTU	OTU	DUB	Ubiquitin	Simkania negevensis	BCV	Chlamydiia	amoeba, humans	M1 only	unknown	transmembrane domain	F8L9T9
24	LotA / Lem21	OTU	DUB	Ubiquitin	Legionella pneumophila	BCV	gammaproteo-bacteria	amoeba, humans	1st domain: K6 only; 2nd: long chains	BCV-surface	two separate catalytic domains	Q5ZTB4
25	LotB / Ceg23	OTU	DUB	Ubiquitin	Legionella pneumophila	BCV	gammaproteo-bacteria	amoeba, humans	K63	BCV-surface, RYK, Rab13, PCYT1A	transmembrane domain	Q5ZV21

26	LotC / Lem27	OTU	DUB	Ubiquitin	Legionella pneumophila	BCV	gammaproteo-bacteria	amoeba, humans	K48>others	VAT1, HMOX1, PPP2R1A		Q5ZSI8
27	LotD / Ceg7	OTU	DUB	Ubiquitin	Legionella pneumophila	BCV	gammaproteo-bacteria	amoeba, humans	promiscuous	unknown		Q5ZYY4
28	SpAID	OTU	DUB	Ubiquitin	Spiroplasma poulsonii	extracellular	Mollicutes	Drosophila melanogaster	unknown	SpAID (auto-active)		A0A2R6Y5P0
29	RickOTU	OTU	DUB	Ubiquitin	Rickettsia massiliae	cytoplasmic	alphaproteo-bacteria	arthropods	K48,K11>others	unknown		A8F1V8
30	BurkOTU	OTU	DUB	Ubiquitin	Burkholderia ambifaria	unknown	betaproteo-bacteria	unknown	K11,K63>others	unknown		B1FBB1
31	EschOTU	OTU	DUB	Ubiquitin	Escherichia albertii	extracellular	gammaproteo-bacteria	vertebrates	K48>others	unknown		B1EF49
32	wPipOTU	OTU	DUB	Ubiquitin	Wolbachia spp.	BCV	alphaproteo-bacteria	Culex pipiens	unknown/weak	unknown	transmembrane domain	A0A8S4QIK8
33	wMelOTU	OTU	DUB	Ubiquitin	Wolbachia spp.	BCV	alphaproteo-bacteria	Drosophila melanogaster	K6>others	unknown		Q73HU7
34	TssM	USP	DUB	Ubiquitin	Burkholderia pseudomalllei	cytoplasmic	betaproteo-bacteria	humans	Ester linkages, also K48, K63, K11	bacterial LPS		D7SFB8
35	SnUSP1	USP	DUB	Ubiquitin	Simkania negevensis	BCV	Chlamydiia	amoeba, humans	promiscuous	unknown		F8L8G5
36	SnUSP2	USP	DUB	Ubiquitin	Simkania negevensis	BCV	Chlamydiia	amoeba, humans	promiscuous, no M1	unknown		F8L8I6
37	SnUSP3	USP	DUB	Ubiquitin	Simkania negevensis	BCV	Chlamydiia	amoeba, humans	K63, K11	unknown		F8L6R0
38	PaJOS	Josephin	DUB	Ubiquitin	Pigmentiphaga aceris	unknown	betaproteo-bacteria	unknown	K63	unknown		A0A5C0B1L0
39	BpJOS	Josephin	DUB (clippase)	Ubiquitin	Burkholderia pyrrocinia	unknown	betaproteo-bacteria	unknown	promiscuous	unknown	leads to Ub destruction	A0A118Q714
40	BcJOS	Josephin	DUB (clippase)	Ubiquitin	Burkholderia catarinensis	unknown	betaproteo-bacteria	unknown	promiscuous	unknown	leads to Ub destruction	A0A1J9PQV4
41	SnJOS1	Josephin	DUB (clippase)	Ubiquitin	Simkania negevensis	BCV	Chlamydiia	amoeba, humans	promiscuous	unknown	leads to Ub destruction	F8L436
42	SnJOS2	Josephin	DUB (clippase)	Ubiquitin	Simkania negevensis	BCV	Chlamydiia	amoeba, humans	promiscuous	unknown	leads to Ub destruction	F8L650
43	PcJOS	Josephin	DUB (clippase)	Ubiquitin	Parachlamydia sp.	BCV	Chlamydiia	amoeba	M1 only	unknown	leads to Ub destruction, transmembrane domain	A0A2H9SU66
44	SnVTD	VTD	DUB	Ubiquitin	Simkania negevensis	BCV	Chlamydiia	amoeba, humans	K6 only	unknown	transmembrane domain	F8L5E7
45	WcVTD1	VTD	DUB	Ubiquitin	Waddlia chondrophila	BCV	Chlamydiia	mammals, amoeba	K6 only	unknown	transmembrane domain	D6YWY5
46	WcVTD2	VTD	DUB	Ubiquitin	Waddlia chondrophila	BCV	Chlamydiia	mammals, amoeba	K63 >> K11	unknown	transmembrane domain	D6YRI4
47	RavD	unclassified	DUB	Ubiquitin	Legionella pneumophila	BCV	gammaproteo-bacteria	amoeba, humans	M1 only	BCV-surface		Q5ZZ51
48	EmcB	unclassified	DUB	Ubiquitin (some Nedd8)	Coxiella burnetii	BCV	gammaproteo-bacteria	vertebrates	K48, K63, K11, K6	RIG-I		Q83A93
49	SopA	bHECT	E3	Ubiquitin	Salmonella enterica	BCV + cytoplasmic	gammaproteo-bacteria	animals	K48	TRIM56, TRIM65 (pro-inflammatory)		Q8ZNR3
50	NleL	bHECT	E3	Ubiquitin	Escherichia coli	extracellular	gammaproteo-bacteria	vertebrates	K6, K48	JNK and ROCK kinases, caspase-4		A0A0H3JDV8
51	PvHECT	bHECT	E3	Ubiquitin	Proteus vulgaris	extracellular	gammaproteo-bacteria	animals	K6, K48	unknown		A0A292CDM3

52	PsHECT	bHECT	E3	Ubiquitin	Pantoea stewartii	extracellular	gammaproteo-bacteria	plants	K48 > K6	unknown		A0ABM6KCL4
53	EaHECT	bHECT	E3	Ubiquitin	Erwinia amylovora	extracellular	gammaproteo-bacteria	plants	unknown	unknown		A0A830ZZW2
54	VsHECT	bHECT	E3	Ubiquitin	Verrucomicrobia spp.	unknown	verruco-microbia	unknown	K48 > K6, K29	unknown		A0A2V2RSR1
55	SspH1	NEL	E3	Ubiquitin	Salmonella enterica	BCV + cytoplasmic	gammaproteo-bacteria	animals	K48	PKN1		D0ZVG2
56	SspH2	NEL	E3	Ubiquitin	Salmonella enterica	BCV + cytoplasmic	gammaproteo-bacteria	animals	K48	NOD1		D0ZPH9
57	SlrP	NEL	E3	Ubiquitin	Salmonella enterica	BCV + cytoplasmic	gammaproteo-bacteria	animals	various	Thioredoxin, DNAJB11		D0ZRB2
58	IpaH1.4	NEL	E3	Ubiquitin	Shigella flexneri	cytoplasmic	gammaproteo-bacteria	humans	K48	HOIP, RNF213		A0A0H2USG1
59	IpaH2.5	NEL	E3	Ubiquitin	Shigella flexneri	cytoplasmic	gammaproteo-bacteria	humans	K48	HOIP		Q2TH08
60	IpaH7.8	NEL	E3	Ubiquitin	Shigella flexneri	cytoplasmic	gammaproteo-bacteria	humans	K48	GSDMB, GSDMD, Glomulin		P18014
61	IpaH4.5	NEL	E3	Ubiquitin	Shigella flexneri	cytoplasmic	gammaproteo-bacteria	humans	K48	p65, TBK1, RPN13, NLRP3		P18009
62	IpaH9.8	NEL	E3	Ubiquitin	Shigella flexneri	cytoplasmic	gammaproteo-bacteria	humans	K48	NEMO, GBP proteins		Q8VSC3
63	YspE1	NEL	E3	Ubiquitin	Yersinia pestis	extracellular	gammaproteo-bacteria	mammals	K48	GBP proteins		A0A3N4BII9
64	YspE2	NEL	E3	Ubiquitin	Yersinia pestis	extracellular	gammaproteo-bacteria	mammals	K48	GBP proteins		A0AAP0VE47
65	RipAR	NEL	E3	Ubiquitin	Ralstonia solanacearum	extracellular	betaproteo-bacteria	plants	degradative chains	plant immune receptor complexes	PTI suppression, K48 inferred from degradative function	A0A7U7JF51
66	RipAW	NEL	E3	Ubiquitin	Ralstonia solanacearum	extracellular	betaproteo-bacteria	plants	degradative chains	plant immune receptor complexes	PTI suppression, K48 inferred from degradative function	Q8XQ11
67	SidC	SNL	E3	Ubiquitin	Legionella pneumophila	BCV	gammaproteo-bacteria	amoeba, humans	K11, K33 > others	Rab10, other Rab GTPases	influence membrane traffic, BCV remodeling	Q5ZSK6
68	SdcA	SNL	E3	Ubiquitin	Legionella pneumophila	BCV	gammaproteo-bacteria	amoeba, humans	unknown	Rab10, other Rab GTPases	influence membrane traffic, BCV remodling	Q5ZSK7
69	SdcB	SNL	E3	Ubiquitin	Legionella pneumophila	BCV	gammaproteo-bacteria	amoeba, humans	unknown	Rab10, other Rab GTPases	influence membrane traffic, BCV remodling	Q5ZSR5
70	AvrPtoB / HopAB	Ubox	E3	Ubiquitin	Pseudomonas syringae	extracellular	gammaproteo-bacteria	plants	degradative chains	Fen kinase, PTI components	PTI suppression, K48 inferred from degradative function	Q8RSY1
71	NleG5-1	Ubox	E3	Ubiquitin	Escherichia coli	extracellular	gammaproteo-bacteria	vertebrates	K48	MED15		Q8X4X3
72	NleG2-3	Ubox	E3	Ubiquitin	Escherichia coli	extracellular	gammaproteo-bacteria	vertebrates	unknown	SNAP29		Q8X509
73	StoD	Ubox	E3	Ubiquitin	Salmonella enterica	BCV + cytoplasmic	gammaproteo-bacteria	animals	unknown	K63 and K48-linked ubiquitin chains	Elongates existing chains	Q8Z7T2
74	GobX	Ubox	E3	Ubiquitin	Legionella pneumophila	BCV	gammaproteo-bacteria	amoeba, humans	unknown	unknown		Q5ZSR2
75	LubX	Ubox	E3	Ubiquitin	Legionella pneumophila	BCV	gammaproteo-bacteria	amoeba, humans	K48	Legionella SidH, host Clk1	meta-effector, transmembrane domain	Q5ZRQ0
76	RavN	Ubox	E3	Ubiquitin	Legionella pneumophila	BCV	gammaproteo-bacteria	amoeba, humans	unknown	unknown		Q5ZWH4
77	SnRING	RING	E3	Ubiquitin	Simkania negevensis	BCV	Chlamydiia	amoeba, humans	K63, K11, K48	unknown		F8L4Z7

78	AnkB / LegAU13	F-box	E3-adaptor	Ubiquitin	Legionella pneumophila	BCV	gammaproteo-bacteria	amoeba, humans	K48	Parvin-B		Q5ZTL7
79	LegU1	F-box	E3-adaptor	Ubiquitin	Legionella pneumophila	BCV	gammaproteo-bacteria	amoeba, humans	K48	host BAT3	transmembrane domain	Q5ZZ40
80	LicA	F-box	E3-adaptor	Ubiquitin	Legionella pneumophila	BCV	gammaproteo-bacteria	amoeba, humans	inactive	unknown	Binds Skp1 but no functional ligase complex	Q5ZVN2
81	VirF	F-box	E3-adaptor	Ubiquitin	Agrobacterium tumefaciens	extracellular	alphaproteo-bacteria	plants	K48	host VFP4, bacterial VirE2	Meta-effector for VirE2 degradation	P15597
82	RipG /GALA	F-box	E3-adaptor	Ubiquitin	Ralstonia solanacearum	extracellular	betaproteo-bacteria	plants	K48	chloroplast proteins, RLCK-VIII-6		Q8XZN9
83	XopL	unclassified	E3	Ubiquitin	Xanthomonas campestris	extracellular	gammaproteo-bacteria	plants	K11	SH3P2, PPI1		Q3BQL2
84	Lug14	unclassified	E3	Ubiquitin	Legionella pneumophila	BCV	gammaproteo-bacteria	amoeba, humans	K11	ARIH2		Q5ZWH9
85	Lug15	unclassified	E3	Ubiquitin	Legionella pneumophila	BCV	gammaproteo-bacteria	amoeba, humans	K6, K33, K48, K63	Sec22b		Q5ZT38
86	Pul-1	unclassified	E3	Ubiquitin	Pseudomonas aeruginosa	extracellular	gammaproteo-bacteria	wide range	promiscous	unknown		Q9I0T2
87	PM19_00185	unclassified	E3	Ubiquitin	Phytoplasma malii	cytoplasmic	Mollicutes	plants	unknown	unknown	transmembrane domain	A0A5B8YVV6
88	TRP120	unclassified	E3	Ubiquitin	Ehrlichia chaffeensis	BCV	alphaproteo-bacteria	mammals	unknown	PCGF5, FBW7, ENO-1		Q2GI62
89	CIF	Cif	Ub/UBL Q40 deamidation	Nedd8, Ubiquitin	Escherichia coli	extracellular	gammaproteo-bacteria	vertebrates	n.a.	Nedd8, Ubiquitin	Deamidation at Q40 interferes with conjugation	Q7WRZ5
90	CHBP	Cif	Ub/UBL Q40 deamidation	Nedd8, Ubiquitin	Burkholderia pseudomalllei	cytoplasmic	betaproteo-bacteria	humans	n.a.	Nedd8, Ubiquitin	Deamidation at Q40 interferes with conjugation	Q63KH5
91	MavC	Cif	Ub Q40-crosslinking	Ubiquitin	Legionella pneumophila	BCV	gammaproteo-bacteria	amoeba, humans	n.a.	Ubiquitin, UBE2N	Crosslink at Q40 interferes with conjugation	Q5ZTL4
92	MvcA	Cif	Ub Q40-unlinking	Ubiquitin	Legionella pneumophila	BCV	gammaproteo-bacteria	amoeba, humans	n.a.	Ubiquitin-UBE2N adduct	Resolves crosslink at Q40	Q5ZTL3
93	OspI	unclassified	UBC-deamidation	none	Shigella flexneri	cytoplasmic	gammaproteo-bacteria	humans	n.a.	UBE2N	Deamidation of UBE2N interferes with K63	Q8VSD5
94	OspG	kinase	Ub-activated kinase	Ubiquitin	Shigella flexneri	cytoplasmic	gammaproteo-bacteria	humans	n.a.	CAND1	E2/ubiquitin-activated kinase activity	Q99PZ6
95	CteC	ART	Ub MARylation	Ubiquitin	Chromobacterium violaceum	cytoplasmic	betaproteo-bacteria	unknown	n.a.	Ubiquitin	Modifies T66 of ubiquitin with ADPR	Q7NY09
96	SidE	SidE	Ub PR-crosslinking	Ubiquitin	Legionella pneumophila	BCV	gammaproteo-bacteria	amoeba, humans	n.a.	Rab GTPases	Installs PR-ubiquitin crosslinks on substrates	Q5ZYX7
97	SdeA	SidE	Ub PR-crosslinking	Ubiquitin	Legionella pneumophila	BCV	gammaproteo-bacteria	amoeba, humans	n.a.	Rab1, Rab33b, Rab6, RTN4B	Installs PR-ubiquitin crosslinks on substrates	Q5ZTK4
98	SdeB	SidE	Ub PR-crosslinking	Ubiquitin	Legionella pneumophila	BCV	gammaproteo-bacteria	amoeba, humans	n.a.	Rab GTPases	Installs PR-ubiquitin crosslinks on substrates	Q5ZTK5
99	SdeC	SidE	Ub PR-crosslinking	Ubiquitin	Legionella pneumophila	BCV	gammaproteo-bacteria	amoeba, humans	n.a.	Rab GTPases	Installs PR-ubiquitin crosslinks on substrates	Q5ZTK8
100	DupA / LaiE	SidE	Ub PR-unlinking	Ubiquitin	Legionella pneumophila	BCV	gammaproteo-bacteria	amoeba, humans	n.a.	PR-ubiquitin adducts	unlinks PR-ubiquitination	Q5ZTK7
101	DupB / SdeD	SidE	Ub PR-unlinking	Ubiquitin	Legionella pneumophila	BCV	gammaproteo-bacteria	amoeba, humans	n.a.	PR-ubiquitin adducts	unlinks PR-ubiquitination	Q5ZSK8
102	LnaB	AMPylase	Ub PR-recovery	Ubiquitin	Legionella pneumophila	BCV	gammaproteo-bacteria	amoeba, humans	n.a.	PR-ubiquitin	restores ubiquitin after PR-ubiquitination	Q5ZSJ0
103	MavL	macro	Ub PR-recovery	Ubiquitin	Legionella pneumophila	BCV	gammaproteo-bacteria	amoeba, humans	n.a.	ADPR-ubiquitin	restores ubiquitin after PR-ubiquitination	Q5ZSJ1
